# Pleiotropic Actions of Forskolin Result in Phosphatidylserine Exposure in Primary Trophoblasts

**DOI:** 10.1371/journal.pone.0081273

**Published:** 2013-12-05

**Authors:** Meghan R. Riddell, Bonnie Winkler-Lowen, Yanyan Jiang, Sandra T. Davidge, Larry J. Guilbert

**Affiliations:** 1 Department of Physiology, University of Alberta, Edmonton, Alberta, Canada; 2 Department of Medical Microbiology and Immunology, University of Alberta, Edmonton, Alberta, Canada; 3 Department of Obstetrics and Gynecology, University of Alberta, Edmonton, Alberta, Canada; 4 Women and Children's Health Research Institute, University of Alberta, Edmonton, Alberta, Canada; University of Cambridge, United Kingdom

## Abstract

Forskolin is an extract of the *Coleus forskholii* plant that is widely used in cell physiology to raise intracellular cAMP levels. In the field of trophoblast biology, forskolin is one of the primary treatments used to induce trophoblastic cellular fusion. The syncytiotrophoblast (ST) is a continuous multinucleated cell in the human placenta that separates maternal from fetal circulations and can only expand by fusion with its stem cell, the cytotrophoblast (CT). Functional investigation of any aspect of ST physiology requires *in vitro* differentiation of CT and *de novo* ST formation, thus selecting the most appropriate differentiation agent for the hypothesis being investigated is necessary as well as addressing potential off-target effects. Previous studies, using forskolin to induce fusion in trophoblastic cell lines, identified phosphatidylserine (PS) externalization to be essential for trophoblast fusion and showed that widespread PS externalization is present even after fusion has been achieved. PS is a membrane phospholipid that is primarily localized to the inner-membrane leaflet. Externalization of PS is a hallmark of early apoptosis and is involved in cellular fusion of myocytes and macrophages. We were interested to examine whether PS externalization was also involved in primary trophoblast fusion. We show widespread PS externalization occurs after 72 hours when fusion was stimulated with forskolin, but not when stimulated with the cell permeant cAMP analog Br-cAMP. Using a forskolin analog, 1,9-dideoxyforskolin, which stimulates membrane transporters but not adenylate cyclase, we found that widespread PS externalization required both increased intracellular cAMP levels and stimulation of membrane transporters. Treatment of primary trophoblasts with Br-cAMP alone did not result in widespread PS externalization despite high levels of cellular fusion. Thus, we concluded that widespread PS externalization is independent of trophoblast fusion and, importantly, provide evidence that the common differentiation agent forskolin has previously unappreciated pleiotropic effects on trophoblastic cells.

## Introduction

A properly formed and well functioning placenta is essential for optimal growth of the fetus and aberrant formation and function of the placenta is associated with the common pregnancy conditions of preeclampsia and intrauterine growth restriction [1]–[3]. The entire surface of the human placenta is covered by a single giant multinucleate cell, the syncytiotrophoblast (ST). The ST is non-proliferative and thus expansion of the ST layer, and therefore the entire placenta, requires fusion and differentiation of the ST stem cell, the cytotrophoblast (CT) [4], [5].

Isolation of ST fragments capable of functioning *in vitro* has not been achieved, thus investigations in to all functional aspects of ST cell physiology requires *de novo* formation of ST in culture through fusion of CT-like cells. The most popular models used to produce syncytium are the choriocarcinoma cell line Bewo and isolated primary CT. Bewo proliferate normally in culture and fuse to form syncytia only upon exogenous stimulation with differentiation agents. Extensive fusion is most commonly stimulated with cell permeant cAMP analogs or the adenylate cyclase activator forskolin for 48 to 72 hours [6], [7]. Fusion results in many of the same differential protein expression patterns seen in primary cells such as production of ST originating hormones (human chorionic gonadotropin (hCG), human placental lactogen) and down regulation of E-cadherin [8]–[10]. In contrast to Bewo cells, primary CT proliferate very slowly in culture and spontaneously fuse to form syncytia [11]. Primary CT fusion has been enhanced by addition of hCG, epidermal growth factor (EGF), GM-CSF, forskolin, or exogenous cAMP analogs such as the cell permeant 8-bromoadensine 3′,5′-cAMP (Br-cAMP) [12]–[14]. Limitations exist for both of these models. For instance, isolation of primary trophoblasts is time consuming, expensive, and difficult and because they are primary cells, transfection and protein knock-down are difficult to achieve. The primary limitation of the Bewo model is their transformed state; thus, key cellular events such as proliferation and differentiation pathways may be altered. Additionally, when exogenous differentiation agents are used in both Bewo and primary CT, potential off target effects of non-specific compounds are possible.

Much is known regarding the molecular pathways controlling trophoblast differentiation, though specific aspects of the differentiation process still require elucidation. The signals that initiate trophoblast differentiation have not been conclusively established but it is thought that when the ST is becoming deficient in intracellular organelles and/or functional nuclei it signals to the CT, which lie directly below the ST [5]. This causes the CT to fuse with the overlying ST, thus becoming incorporated in the ST and becoming a functional part of the syncytium. Several fusogenic proteins have been identified to be involved in trophoblast fusion including the endogenous retroviral proteins Syncytin-1 and Syncytin-2 [10], [13], [15].

Another apparently obligate step that has been identified for trophoblast fusion is the externalization of the membrane phospholipid phosphatidylserine (PS) [9], [16]. PS externalization has been shown to be involved in fusion of both myoblasts and macrophages with a transient exposure of PS at contact areas between two fusing cells [17]–[19]. In trophoblastic cell lines PS externalization was observed to cover essentially the entire surface of fused cells and persisted after fusion was achieved [9], [16], [20]. Cell fusion was blocked by ∼40% in the JAR trophoblastic cell line stimulated to fuse with forskolin, using one monoclonal anti-PS antibody, though another monoclonal anti-PS antibody had no effect on fusion [16].

PS efflux is an important event in cellular apoptosis, externalization of PS in platelets is involved in the initiation of the clotting cascade, and non-apoptotic PS externalization has been observed in cardiomyocytes [21], [22]. PS externalization is controlled by the activation of an as of yet unidentified membrane protein or group of membrane proteins known as scramblase(s). Activation of scramblase activity can be stimulated by increases in cytoplasmic Ca^2+^ or apoptotic pathway mediators (though which mediators are involved remains to be identified) [21]. In cardiomyocytes PS externalization can also be induced by the inhibition of flippase [22]. Flippases are ATP-dependent membrane proteins, such as aminophospholipid translocase, which transport the negatively charged aminophospholipids PS and phosphatidylethanolamine to the inner membrane leaflet and maintain normal membrane asymmetry [21]–[23]. Thus, it is likely the combination of the activation of scramblase(s) and the inhibition of flippase activity that lead to PS exposure on the outer membrane.

As noted previously, PS externalization is considered to be an obligatory event for trophoblast fusion, however this conclusion is based on data produced in trophoblastic cell lines such as Bewo and have never been validated with primary cells. Therefore, we sought to examine whether PS externalization was required for fusion in primary trophoblasts.

## Materials and Methods

### Cells, Tissues and Ethical Approval

The Bewo choriocarcinoma cell line was obtained from the American Type Culture Collection (ATCC, Rockville, USA). Primary trophoblasts were isolated from normal term placentas at 37-39 weeks gestation that were delivered by caesarean section without labour after written informed consent was obtained and full ethics review by the University of Alberta Ethics Committee. Purified villous CT were isolated by trypsin-DNAse digestion of minced chorionic tissue and immunoabsorbtion onto immunoglobulin (Ig)-coated glass bead columns as previously described [11] using antibodies to CD9 (clone 50H.19, house preparation), major histocompatibility complex (MHC) class I (clone W6/32, Harlan Sera-Lab, Crawley Down, Sussex, UK) and MHC class II (clone 7H3, house preparation) then cryopreserved in fetal calf serum (FCS, Gibco, Grand Island, NY) made 10% in dimethylsulfoxide (DMSO; Sigma, Oakville, ON) in liquid nitrogen. After seeding onto tissue culture plastic, <0.01% of the population were mesenchymal cells by vimentin staining as previously described [11].

### Primary Cell Culture

CT were seeded at a density of 1×10^5^ cells per well in 96 well plates (Nunc, Roschester, NY) in Iscoves Modified Dulbecco's Medium (IMDM, Gibco, Grand Island, NY) supplemented with 10% FCS (Gibco) (IMDM-FCS) and antibiotics (end concentration penicillin 100 U/ml, streptomycin 100 µg/ml; Sigma) and incubated for 4 hours under a fully humidified standard atmosphere. The cells were then washed to remove non-adherent cells and medium was changed to either IMDM-FCS or IMDM-FCS plus 10 µM of the cell permeable cAMP analog Br-cAMP (cAMP; Sigma), 10 µM forskolin (Sigma), 10 µM 1,9-dideoxyforskolin (Sigma), the combination of cAMP plus 1,9-dideoxyforskolin, or solvent control DMSO in triplicate wells per treatment. Cells were then returned to the incubator for 24, 48, or 72 hours and the medium was changed after 48 hours.

### Bewo Cell Culture

Bewo cells were maintained in Nutrient Mixture F12 Hams (Gibco) supplemented with 15% FCS (Gibco) (Hams-FCS) and antibiotics (end concentration penicillin 100 U/ml, streptomycin 100 µg/ml; Sigma) in 6 well dishes (Corning, Lowell, MA). When 75-85% confluent the cells were passaged using 0.05% trypsin-EDTA (Gibco) for 5 minutes and then seeded into 96 well plates at a density of 10,000 cells per well in Hams-FCS. Cells were incubated for 4 hours in a fully humidified standard atmosphere and medium was changed to Ham-FCS with or without 10 µM forskolin, 10 µM 1,9-dideoxyforskolin, 250 µM Br-cAMP, both Br-cAMP and 1,9-dideoxyforskolin, or solvent control DMSO in triplicate wells per treatment. Medium was changed after 48 hours and cells were incubated for a total of 72 hours. Lower concentrations of Br-cAMP were tested (10–100 µM) but were not found to stimulate statistically significant rates of fusion (30% fusion or less).

### Assessment of Cell Fusion

Primary trophoblasts were fixed after 24, 48, or 72 hours in culture with methanol and stained for the cellular junction protein desmoplakin as previously described [14], [24].

Bewo cellular fusion was assessed with E-cadherin antibody. After 72 hours in culture cells were fixed with methanol for 10 minutes followed by a one hour incubation with 20% normal goat serum (Gibco) and then incubated overnight at 4°C with anti-E-cadherin (5 µg/mL;Biolegend; San Diego, CA) or the appropriate non-immune control. The cells were then washed and incubated with Alexafluor goat anti-mouse 488 (Invitrogen, Burlington, ON) for one hour and the nuclei were visualized with DAPI (3 µM; Research Organics, Cleaveland, OH) for 10 min at room temperature. Triplicate images were randomly captured per well on an Olympus IX2-UCB microscope equipped with a Roper Scientific camera and a Sutter Instruments Lambda DG-4 fluorescent lamp (Olympus, Melville, NY). Slidebook 3.0 (Carsen, Markham, ON, Canada) was utilized as capture software and Image-J for analysis. The percentage of nuclei lacking expression of E-cadherin were assessed, since E-cadherin expression has been shown to decrease with differentiation into syncytium [9].

### Annexin-V Binding

Both primary trophoblasts and Bewo were washed once in annexin-V binding buffer (10 mM HEPES, 140 mM NaCl, 2.4 mM CaCl_2_; pH 7.4) then annexin-V-FITC (Biolegend, San Diego, CA) was added in annexin-V binding buffer (1∶50 for primary cells and 1:25 for Bewo) and the cells were incubated for 1 hour in an incubator under standard conditions. Cells were then washed twice with annexin-V binding buffer and fixed with 4% paraformaldehyde for 10 min. Nuclei were visualized with DAPI and triplicate images of each well were randomly obtained as detailed above. The number of nuclei associated with areas of annexin-V binding were counted. As a positive control primary trophoblasts and Bewo were incubated with 0.3 µM staurosporine for 4 hours and then annexin-V binding was visualized to establish the amount of annexin-V required to visualize PS externalization (see Figure S1).

### Statistical Analysis

All experiments were performed a minimum of three times with experiments utilizing primary trophoblasts carried out on cells isolated from at least 3 different pregnancies. Details of the statistical analysis carried out for each figure can be found in the figure legends. All statistics were carried out using Prism 5.0 software and a p<0.05 was considered significant.

## Results

**Figure 1:**
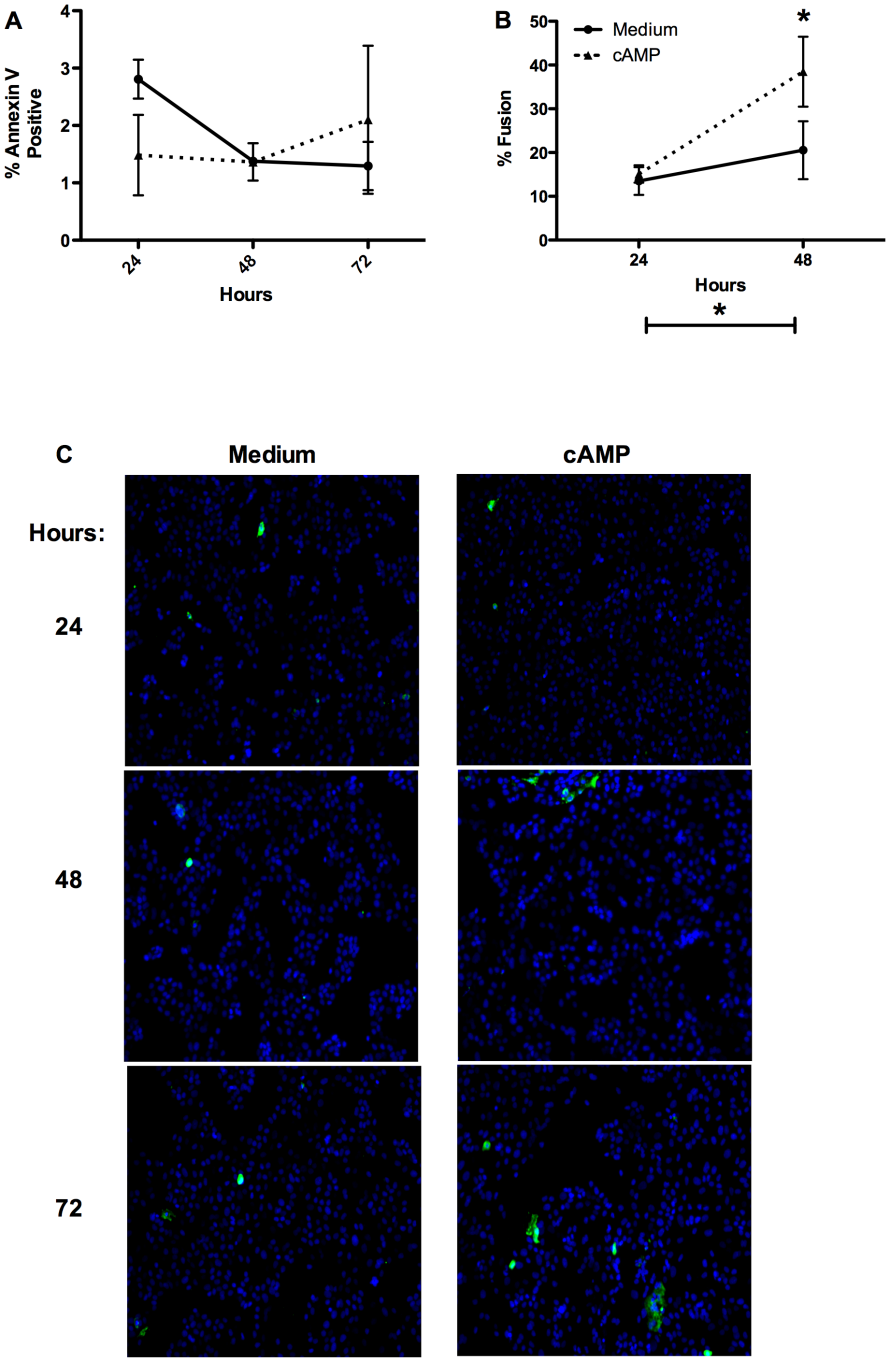
PS externalization and cellular fusion in primary trophoblasts treated with cAMP. A) Summary graph of cellular fusion after 24 and 48 hours; n = 3; repeated measures two-way ANOVA with Bonferroni post hoc analysis; * = p<0.05; B) Summary graph of the proportion of cells with exposed PS measured with annexin-V-FITC analyzed in parallel wells of primary CT to those analyzed for fusion; C) Representative images of annexin-V-FITC binding in primary CT.

In order to examine whether expansive PS externalization was occurring in primary trophoblasts during the differentiation process, as was observed in Bewo cell line, cells were probed with annexin-V-FITC. We have previously shown that the spontaneous fusion rate of primary trophoblasts after 72 hours is ∼30% and that treatment with Br-cAMP (cAMP) for the same time period leads to fusion of ∼75% the cells [14]. Here we found that the rates of CT fusion rose significantly by 48 hours in both spontaneously fusing and cAMP treated cells (to ∼20% in spontaneously fusing cells and ∼30% in cAMP treated) and cAMP significantly increased fusion over medium alone after 48 hours (Figure 1b). In parallel wells, PS externalization measured with annexin-V was not found to significantly rise at 24, 48, or 72 hours with or without cAMP treatment (Figure 1a; representative annexin-V binding images Figure 1c). As widespread PS externalization has previously been shown only in trophoblastic cell lines stimulated to fuse with forskolin after 72 hours [9], [16], [20] we went on to treat primary CT with forskolin.

**Figure 2:**
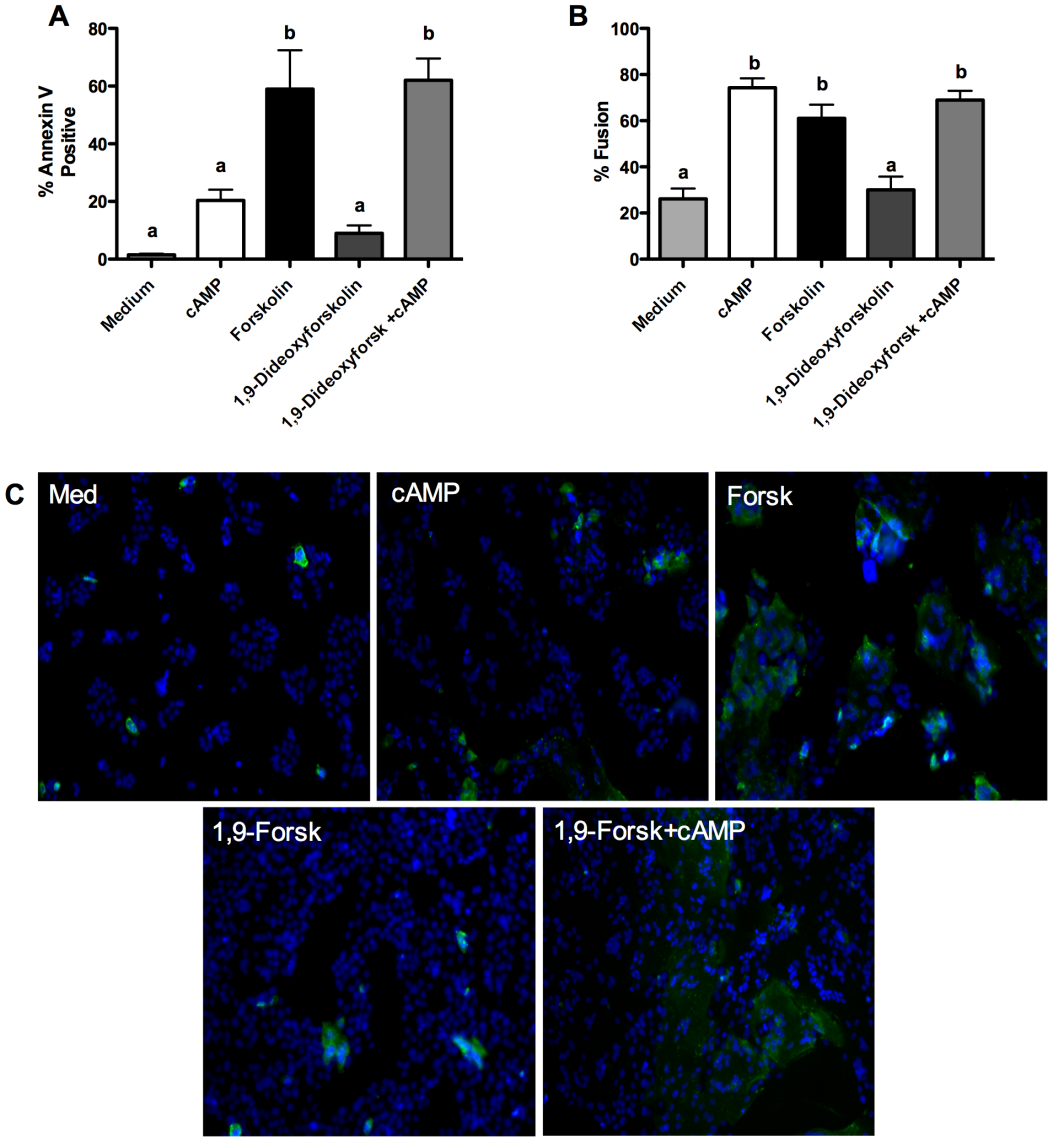
PS externalization and cellular fusion in primary trophoblasts treated with cAMP, forskolin or 1,9-dideoxyforskolin. A) Summary graph of annexin-V positive cells after 72 hours; n = 3; mean+/− SEM; one-way ANOVA with Bonferroni post-hoc analysis; a = p<0.05 vs. medium, cAMP, and 1,9-dideoxyforskolin; B) Summary graph of cellular fusion after 72 hours; n = 3; mean +/−SEM; one-way ANOVA with Bonferroni post-hoc analysis; b = p<0.01 vs. medium and 1,9-dideoxyforskolin; C) Representative images of annexin-V-FITC binding after 72 hours; Forsk =  forskolin; 1,9-Forsk =  1,9-dideoxyforskolin.

**Figure 3:**
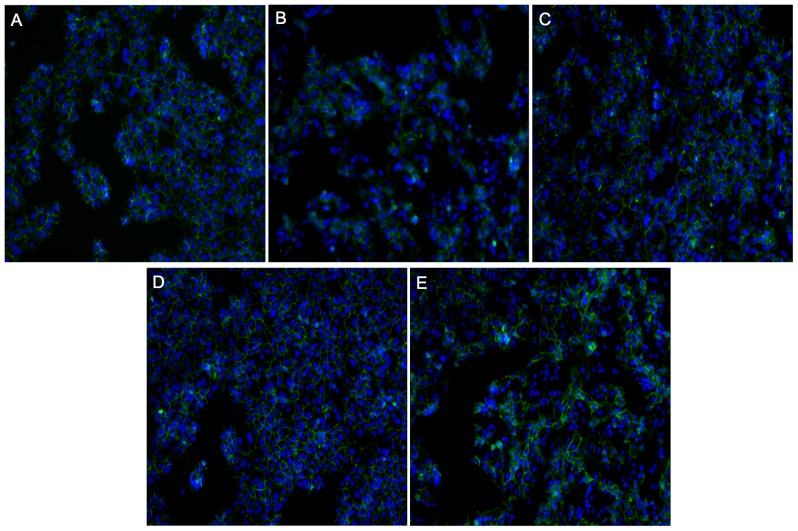
Representative images of desmoplakin staining after 72 hours in culture used to determine fusion levels in primary trophoblasts. Green = desmoplakin; Blue =  nucleus; arrow heads indicate areas of fusion; A) medium alone; B) cAMP; C) forskolin; D) 1,9-dideoxyforskolin; E) cAMP and 1,9-dideoxyforskolin.

In contrast to treatment with cAMP, treatment of primary CT with forskolin for 72 hours led to fusion of ∼60% of nuclei concomitant with the same proportion of cells having externalized PS (Figure 2a, 2b). Since forskolin treatment and addition of cAMP stimulate trophoblast fusion through seemingly similar mechanisms (increased intracellular cAMP concentrations) but result in very different patterns of PS externalization, we went on to examine whether this may be due to pleiotropic effects of forskolin beyond adenylate cyclase activation. Forskolin is known to modulate voltage gated K^+^ channels, to inhibit the glucose transporter GLUT1 and interact with ATP-binding cassette sub-family member B1 (ABCB1) in addition to modulating adenylate cyclase activity [25]–[27]. An analog of forskolin, 1,9-dideoxyforskolin, does not stimulate adenylate cyclase activation but interacts with and modulates the above membrane transporters [25]–[27]. Thus we utilized 1,9-dideoxyforskolin to understand whether the non-adenylate cyclase effects of forskolin may be causing the observed increased proportion of cells with externalized PS. 1,9-dideoxyforskolin treatment for 72 hours did not significantly stimulate PS externalization or trophoblast fusion (Figure 2a, 2b; representative fusion image Figure 3d). In combination with cAMP 1,9-dideoxyforskolin stimulated high levels PS externalization (∼60% positive nuclei) and trophoblast fusion (∼70%) (Figure 2a, 2b; representative fusion image Figure 3e).

**Figure 4:**
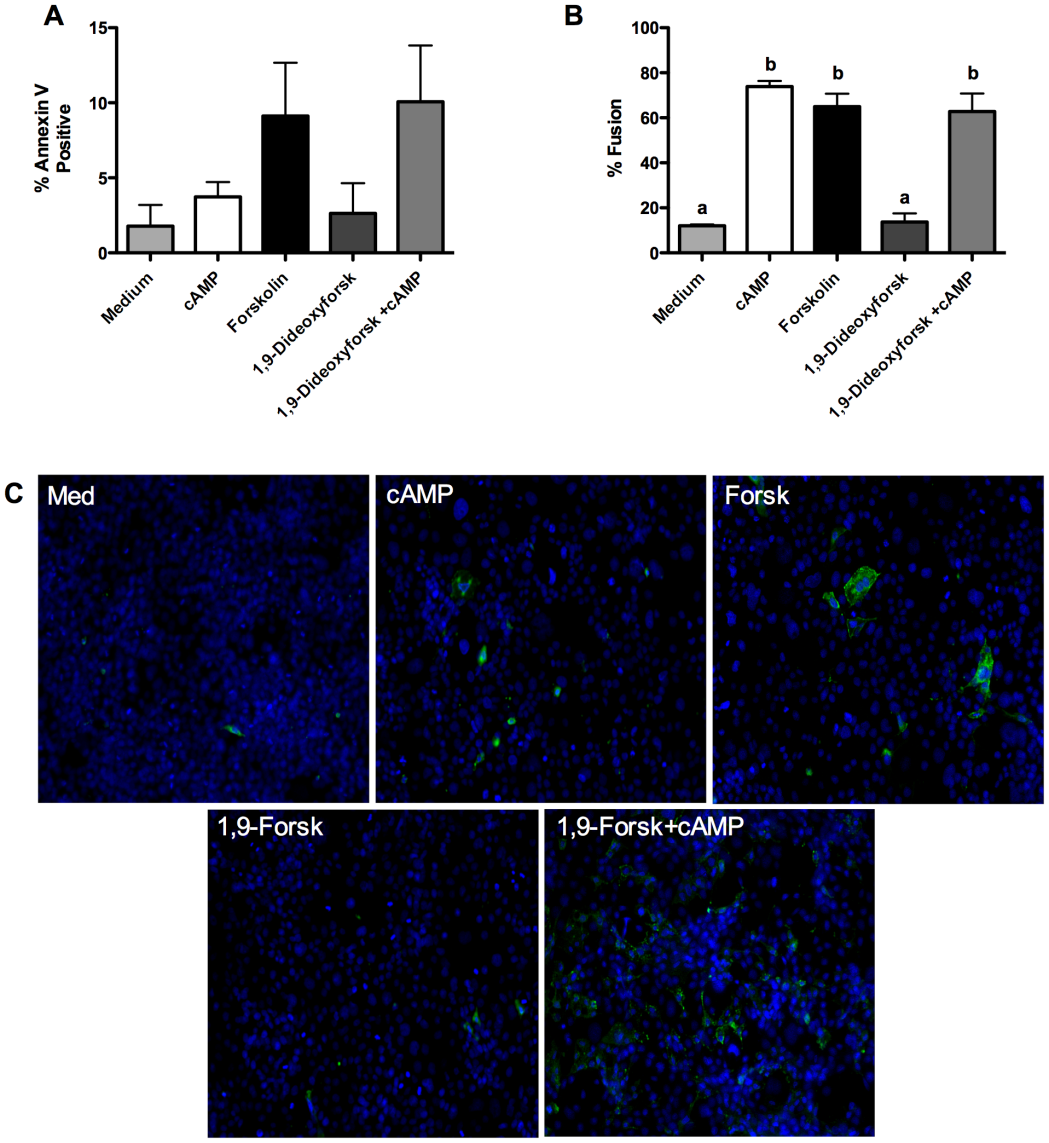
Externalized PS levels are low in Bewo despite high amounts of cellular fusion. A) Summary graph of annexin-V positive cells after 72 hours; n = 4; mean+/− SEM; one-way ANOVA with Bonferroni post-hoc analysis; B) Summary graph of cellular fusion after 72 hours; n = 4; mean +/−SEM; one-way ANOVA with Bonferroni post-hoc analysis; b = p<0.01 vs. medium and 1,9-dideoxyforkolin; C) Representative images of annexin-V-FITC binding after 72 hours; Forsk =  forskolin; 1,9-Forsk =  1,9-dideoxyforskolin.

**Figure 5:**
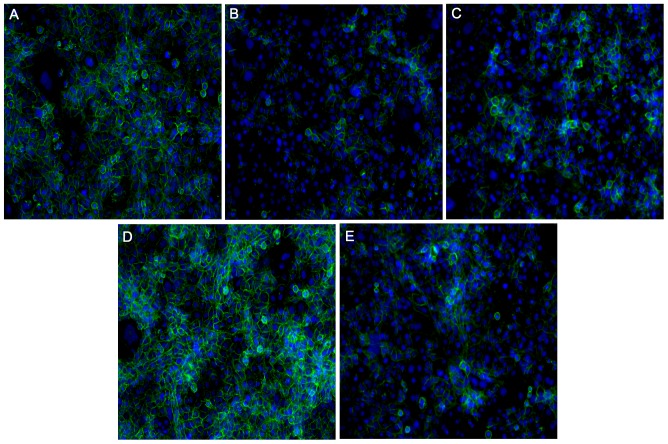
Representative images of E-cadherin staining after 72 hours in culture used to determine fusion levels in Bewo. Green =  E-cadherin; Blue =  nucleus; A) medium alone; B) cAMP; C) Forskolin; D) 1,9-dideoxyforskolin; E) cAMP and 1,9-dideoxyforskolin.

Treatment of Bewo choriocarcinoma cells with cAMP stimulated cellular fusion (∼70%), but did not stimulate high levels of PS externalization (∼4%) (Figure 4a, 4b; representative fusion image Figure 5b). Forskolin stimulated widespread cellular fusion (∼65%), but PS externalization was limited (∼10%) in the cell line, though high variability was observed in the amount of PS externalization (representative fusion image Figure 5c). Treatment with 1,9-dideoxyforskolin alone did not significantly stimulate cellular fusion or PS externalization. High levels of cellular fusion were observed when Bewo were treated with both cAMP and 1,9-dideoxyforskolin and the proportion of cells with externalized PS rose (to ∼10%), but not significantly above medium alone. Again, as with forskolin treatment, a very high variability was observed in the proportion of cells with externalized PS with this treatment.

## Discussion

In this study we examined whether PS externalization was involved in primary villous CT differentiation into syncytium. We found that extensive PS externalization only occurs when differentiation is stimulated with the adenylate cyclase activator forskolin or the combination of cAMP and the forskolin analog 1,9-dideoxyforkolin, and that cAMP treatment alone and spontaneous trophoblast fusion do not result in significant extensive PS externalization in primary trophoblasts. Therefore extensive PS externalization is likely due to modulation of membrane transporters by forskolin and its analog, which does not stimulate adenylate cyclase activity, and is also a process that requires increased intracellular cAMP concentrations. Our data also demonstrated that extensive PS externalization is independent of trophoblast fusion in primary trophoblasts.

We also assessed Bewo cells and found that the proportion of cells that were observed to have externalized PS was much lower than the proportion of fused cells observed and that extensive PS externalization was not consistently observed. Previously, using data produced in trophoblastic cell lines stimulated to fuse with forskolin, it was established that PS externalization on the entire surface of syncytium occurred concomitant with cellular fusion and that a monoclonal anti-PS antibody was capable of inhibiting cellular fusion [9], [16], [20]. The authors concluded based on this work that PS externalization was required for trophoblast fusion. This conclusion is in contrast with ours but a direct comparison of the data are not available as the previous publications do not contain a summary of the proportion of cells that are positive for externalized PS. Further, the authors also do not show an increase in PS externalization over a medium alone control after forskolin treatment [9], [20]. Additionally images of positive staining are not presented or limited to clusters of fewer than 10 nuclei, thus making comparison to our experiments difficult [9], [20].

Forskolin and 1,9-dideoxyforskolin are known to interact with and modulate activity of voltage gated K^+^ channels, the glucose transporter GLUT1, and ABCB1 (MDR1) [25]–[27]. Since the exact transporters responsible for PS externalization remain to be elucidated, it is possible that forskolin and 1,9-dideoxyforskolin stimulate one or more of these unidentified transporters directly or cause the inhibition of flippases, which maintain normal phosphlipid membrane asymmetry [21]–[23]. If a direct interaction is occurring it, appears to be insufficient to cause PS externalization without increased levels of intracellular cAMP. The expansive and extended externalization of PS in primary trophoblasts appears to require the combined effects of forskolin: the stimulation or inhibition of unknown transporters and increased intracellular cAMP. In particular, 1,9-dideoxyforskolin is not capable of stimulating expansive PS externalization without the addition of exogenous cAMP. Expansive PS externalization was inconsistently observed in the Bewo cell line but the trend towards increased PS efflux with forskolin and the combined treatment of 1,9-dideoxyforskolin plus cAMP was observed. Importantly, Bewo required a cAMP concentration 25 times higher than primary cells for significant fusion to be observed; thus it appears Bewo are far less sensitive to exogenous cAMP treatment than primary cells. This decreased sensitivity of Bewo cells to cAMP in turn may be related to the inconsistent effects observed on Bewo externalization of PS. Since it has been previously shown in trophoblastic cell lines that PS externalization during forskolin induced differentiation is not associated with increased levels of apoptosis, such externalization appears to be an apoptosis-independent event and forskolin may be useful in answering fundamental questions about what proteins and cellular pathways are involved in PS externalization under non-apoptotic conditions.

Forskolin is used extensively in the trophoblast literature as a differentiation agent of trophoblastic cell lines though, to our knowledge, the pleiotropic effects of forskolin beyond activation of adenylate cyclase have not been widely, if ever, acknowledged. Use of forskolin as a differentiating agent for Bewo was initially presented by Wice et al. (1990) where only the adenylate cyclase activating properties of forskolin are discussed. The data presented here demonstrates that forskolin can have pleiotropic effects on trophoblastic cells independent of adenylate cyclase activity.

Though it appears that extensive PS externalization is not involved in trophoblastic cell fusion, our experiments have not directly examined whether transient PS externalization at the site of membrane fusion is occurring in trophoblasts. This pattern of PS externalization has been observed in myoblast and macrophage fusion [17]–[19]. Since the exposure of PS at the actual site of membrane fusion could be very short lived, the large disparity in the numbers between cells that will fuse (either spontaneously or with cAMP treatment) and those that were observed to bind annexin-V does not rule out that this may be occurring. Additionally Adler et al. (1995) have shown in the JAR choriocarcinoma cell line that one monoclonal anti-PS antibody (though not another monoclonal anti-PS antibody) can inhibit forskolin-induced fusion by 40%. Similarly designed experiments using anti-PS antibodies and annexin-V have been shown to block myoblast formation [18], [19]. Thus close examination of phosphlipid membrane localization warrants further investigation in trophoblasts to completely exclude the involvement of externalized PS in the fusion process.

Ultimately the data presented in this communication suggest that PS externalization is unlikely to be involved in trophoblastic cell fusion and brings to the attention of the reader the pleiotropic effects of forskolin treatment on trophoblastic cells.

## Supporting Information

Figure S1 Representative images of annexin-V-FITC binding to trophoblastic cells after induction of apoptosis with staurosporine. A) Primary trophoblasts 4 hours after staurosporine treatment; B) Bewo cells 4 hours after staurosporine treatment.
(Tif)
