# Supplementary figures and images for: Pleiotropic Actions of Forskolin Result in Phosphatidylserine Exposure in Primary Trophoblasts
Source: PLoS One. 2013 Dec 5;8(12):e81273. doi: 10.1371/journal.pone.0081273 (PMC3855289; doi:10.1371/journal.pone.0081273)

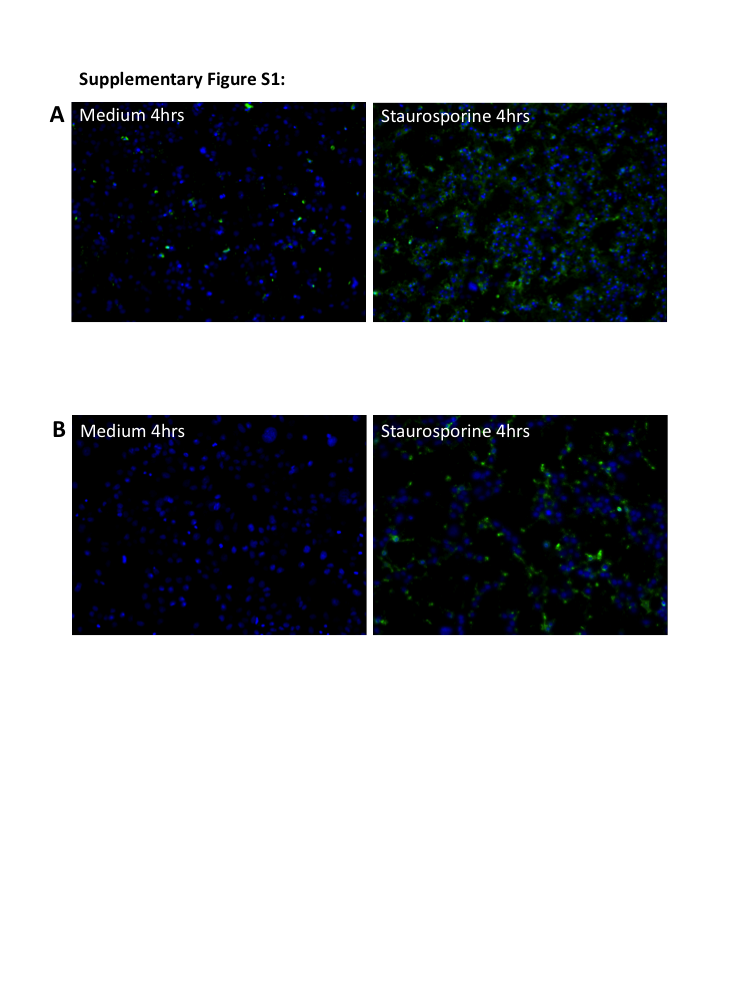

Supplement: Figure S1 — Representative images of annexin-V-FITC binding to trophoblastic cells after induction of apoptosis with staurosporine. A) Primary trophoblasts 4 hours after staurosporine treatment; B) Bewo cells 4 hours after staurosporine treatment. (Tif) [file pone.0081273.s001.tif]
